# P-1953. Incidence, Clinical Characteristics, and Outcomes of Candida Endocarditis in Patients with Candidemia

**DOI:** 10.1093/ofid/ofaf695.2121

**Published:** 2026-01-11

**Authors:** Molly C Studebaker, Isabel C Campa, Andrés F Henao Martínez, Martin Krsak, Daniel B Chastain

**Affiliations:** University of Georgia College of Pharmacy, Moultrie, GA; University of Georgia College of Pharmacy, Moultrie, GA; University of Colorado Anschutz Medical Campus, Aurora, Colorado; University of Colorado School of Medicine, CO; University of Georgia College of Pharmacy, Moultrie, GA

## Abstract

**Background:**

Candida endocarditis (CE) is a severe complication of candidemia with a poor prognosis. Despite its clinical importance, data on the incidence and risk factors for CE remain limited. This study aimed to characterize patients with CE and identify factors associated with its occurrence.

**Table.**
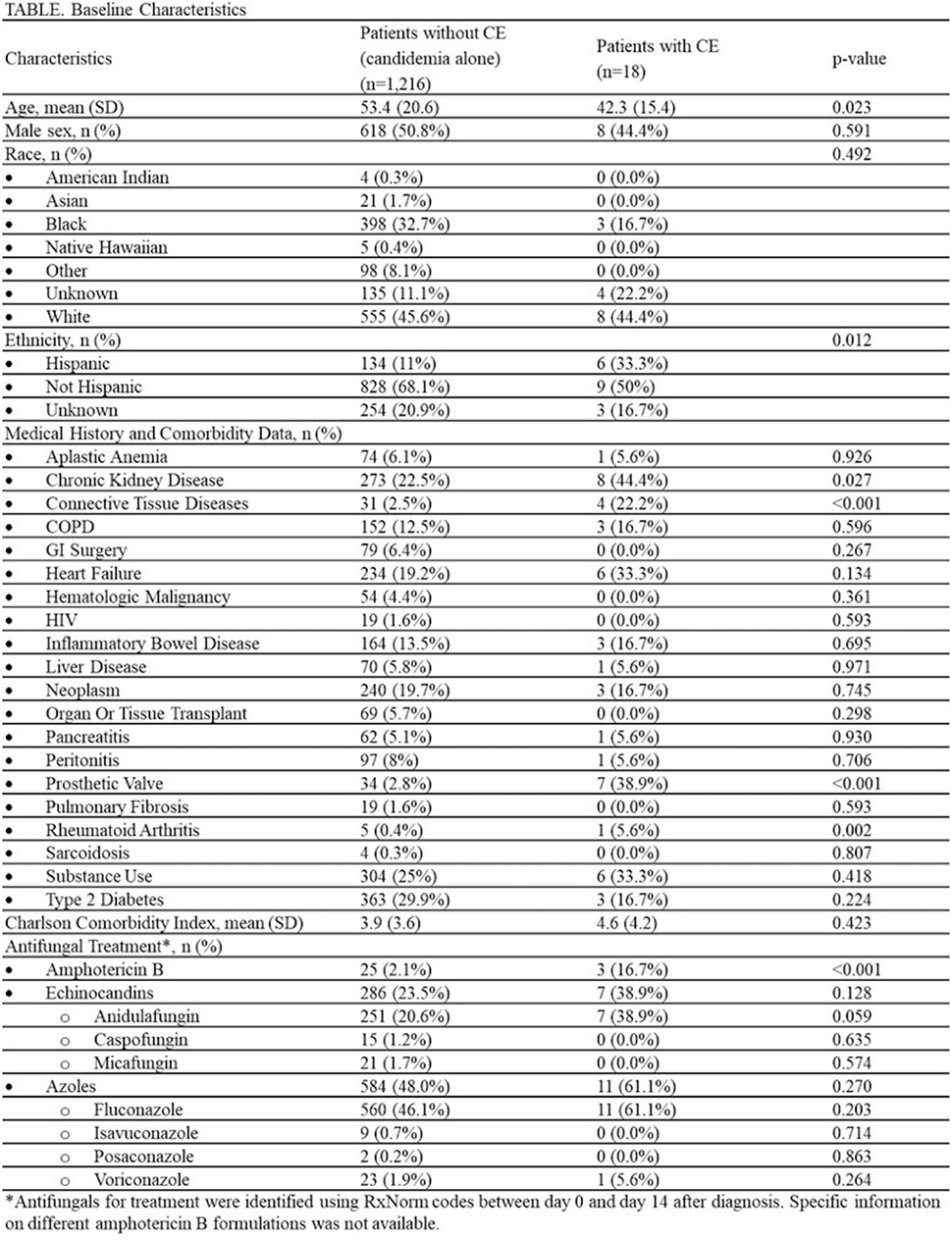
Baseline Characteristics *Antifungals for treatment were identified using RxNorm codes between day 0 and day 14 after diagnosis. Specific information on different amphotericin B formulations was not available.

**Figure.**
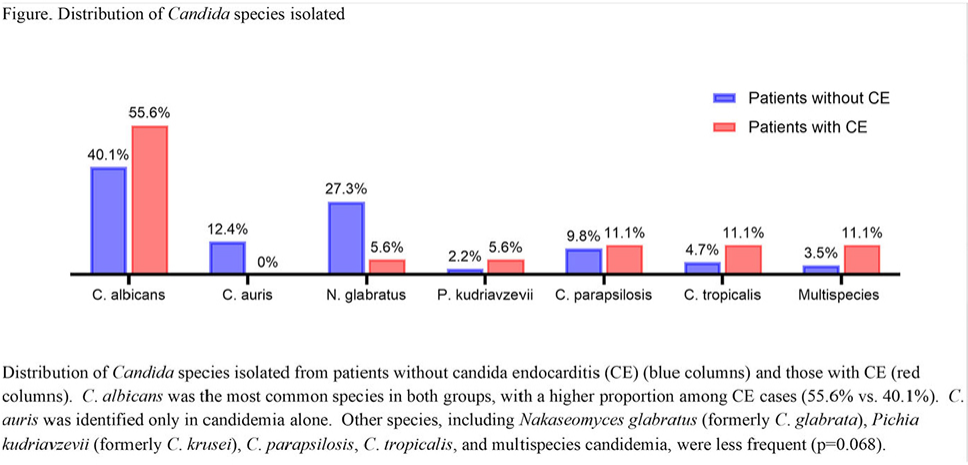
Distribution of Candida species isolated Distribution of Candida species isolated from patients without candida endocarditis (CE) (blue columns) and those with CE (red columns). C. albicans was the most common species in both groups, with a higher proportion among CE cases (55.6% vs. 40.1%). C. auris was identified only in candidemia alone. Other species, including Nakaseomyces glabratus (formerly C. glabrata), Pichia kudriavzevii (formerly C. krusei), C. parapsilosis, C. tropicalis, and multispecies candidemia, were less frequent (p=0.068).

**Methods:**

We performed a retrospective cohort study using TriNetX, a global federated health research network. Adults (≥ 18 years) with candidemia, confirmed by blood PCR testing between May 2017 and November 2023, were included. CE was defined by the presence of ICD-10 code B37.6. Patients were categorized as having candidemia alone or CE. Demographics, comorbidities, *Candida* species, antifungal therapy, and 1-year mortality were compared between groups.

**Results:**

Of 1234 patients with candidemia, 18 (1.5%) were diagnosed with CE. Patients with CE were younger (mean age 42.3 vs 53.4 years, p=0.023) and more likely to identify as Hispanic (33.3% vs 11%, p=.012) (Table). Prosthetic valves (38.9% vs 2.8%, p< .001), systemic connective tissue disease (22.2% vs 2.5%, p< 0.001), and chronic kidney disease (44.4% vs 22.5%, p=0.027) were significantly more common in CE. *C*. *albicans* was the most frequently isolated species in both groups, though more common in CE (55.6% vs 40.1%, p=0.068) (Figure). Patients with CE were more likely to receive an echinocandin (38.9% vs. 23.5%; p=0.128) or amphotericin B (16.7% vs. 2.1%; p< 0.001), while azole antifungal use was similar between groups (50% vs. 29.8%; p=0.063), with fluconazole being the most commonly used azole in both. One year mortality was comparable between groups (27.8% for CE vs. 27.5% for candidemia alone; p=0.976).

**Conclusion:**

In this large candidemia cohort, CE was rare but associated with identifiable risk factors, including prosthetic heart valves, systemic connective tissue disease, and chronic kidney disease. Although *C*. *albicans* was more frequently isolated in CE cases, overall, *Candida* species distribution was similar. Despite differences in clinical characteristics and antifungal therapy, 1-year mortality did not differ between patients with and without CE.

**Disclosures:**

Andrés F. Henao Martínez, MD, MPH, F2: Grant/Research Support|Scynexis: Grant/Research Support

